# A systematic literature review of knowledge graph construction and application in education

**DOI:** 10.1016/j.heliyon.2024.e25383

**Published:** 2024-02-01

**Authors:** Bilal Abu-Salih, Salihah Alotaibi

**Affiliations:** a King Abdullah II School of Information Technology, The University of Jordan, Amman, Jordan; b Information Systems Department, College of Computer and Information Sciences, Imam Mohammad Ibn Saud Islamic University (IMSIU), 11432, Riyadh, Saudi Arabia

**Keywords:** Knowledge graphs, Knowledge graph construction, Education, Learning, Systematic literature review, Survey

## Abstract

In the dynamic landscape of modern education, the search for improved pedagogical methods, enriched learning experiences, and empowered educators remains a perpetual pursuit. In recent years, a remarkable technological innovation has asserted its dominance in education: Knowledge Graphs (KGs). These structured representations of knowledge are increasingly proving to be indispensable tools, fostering advancements driven by the growing recognition of their essential role in enriching personalised learning, curriculum design, concept mapping, and educational content recommendation systems. In this paper, a systematic literature review (SLR) has been conducted to comprehensively examine KG construction methodologies and their applications across five key domains in education. In each examined study, we highlight the specific KG functionalities, knowledge extraction techniques, knowledge base characteristics, resource requirements, evaluation criteria, and limitations. This paper distinguishes itself by offering a broad overview of KGs in education, analyzing state-of-the-art methodologies, and identifying research gaps and limitations, paving the way for future advancements.

## 1 Introduction

In the dynamic landscape of modern education, there is a constant pursuit of enhancing pedagogical methods, empowering educators, and ultimately enriching students' learning experiences. Over recent years, one remarkable technological advancement has steadily asserted its prominence in education: Knowledge Graphs (KGs). These structured representations of knowledge have emerged as indispensable tools in education, catalysing advances driven by their increasingly recognized vital role in augmenting personalised learning, curriculum design, concept mapping, and educational content recommendation systems [[1], [2], [3], [4]]. KGs are not a new concept; they have been utilized extensively in domains like healthcare [5], finance [6], transportation [7], business [8], and other domains [[9], [10], [11], [12], [13], [14]]. However, their robust integration into the educational realm is a testament to their transformative potential. KGs, in the context of education, signify an interconnected web of information, concepts, and relationships meticulously structured to reflect the diverse facets of educational content and pedagogical practices [15,16].

This paper aims to carry out a Systematic Literature Review (SLR) to comprehensively analyze and synthesize the multifaceted applications of KGs in the education domain. The motivation behind this endeavour is the compelling and accelerating wave of innovative developments, driven by the growing realization that KGs have the capacity to revolutionize traditional educational paradigms [17,18]. Throughout this review, we navigate the ever-expanding body of scholarly work, encompassing studies, experiments, and applications that harness the power of KGs to push education forward. This SLR encapsulates the remarkable advancements and challenges encountered along the way. The heart of this exploration lies in Five primary domains: Adaptive and Personalised Learning, Curriculum Design and Planning, Concept Mapping and Visualization, Semantic Search and Questioning Answering, and other relevant miscellaneous applications. These domains serve as the compass guiding us through the diverse and enriching landscape of educational KG applications. In each of these realms, KGs offer the promise of tailored, data-driven, and adaptive educational experiences. In this context, we thoroughly examine these methodologies, providing concise summaries for each approach. These summaries elucidate the specific functionalities of the constructed KG, the techniques employed for knowledge extraction and construction, the nature of the knowledge bases, the requisite resources for KG construction, pertinent statistics about the KGs, the criteria used to evaluate KG construction methods, as well as their respective limitations and drawbacks. This paper distinguishes itself from related works, which often possess a more limited scope [19,20] or focus on a cross-domain application of education [21]. Our contributions stand out in several ways.

• To the best of our knowledge, this SLR represents the first comprehensive overview of KG construction and application within the realm of education.
• We conduct an in-depth analysis of cutting-edge KG construction methodologies, offering a balanced assessment of their strengths and weaknesses.
• We encapsulate the limitations and inadequacy of the current approaches, thereby identifying areas for further research and charting a course for future exploration.

The remainder of this paper is organised as follows: Section 2 shows the methodology followed in the SLR. Section 3 discusses the notion of domain-specific KG and its significance within education from multiple vantage points. Section 4 details various KG construction approaches pertinent to diverse educational domains. Section 5 aggregates the research issues in the literature, and Section 6 synthesises predominant trends in existing techniques, highlights research gaps, and furnishes recommendations to address these gaps effectively. Section 7 concludes the paper.

## 2 Methodology

This paper aims to conduct a systematic literature review (SLR) to explore recent KG construction approaches and their applications in the context of the education domain. The objective is to comprehensively cover papers describing mechanisms for KG construction to enhance educational applications. This SLR focuses on articles published within the past five years (2019–2023) to ensure relevance and currency. The review adheres to the PRISMA (Preferred Reporting Items for Systematic Reviews and Meta-Analyses) framework for guidance [22]. To assemble the initial set of articles, an extensive search was conducted across various databases, including but not limited to Elsevier, ACM Digital Library, Multidisciplinary Digital Publishing Institute (MDPI), IEEE Xplore digital library, and Google Scholar. The search was limited to English-language articles and employed a set of keywords in the query, such as "Knowledge Graph Construction", "Education", "E-Learning", "Personalised Learning", “Adaptive Learning”, "Concept Mapping", "Curriculum Design", "Semantic Search", "Question Answering," and various other related terms.

**Fig. 1:**
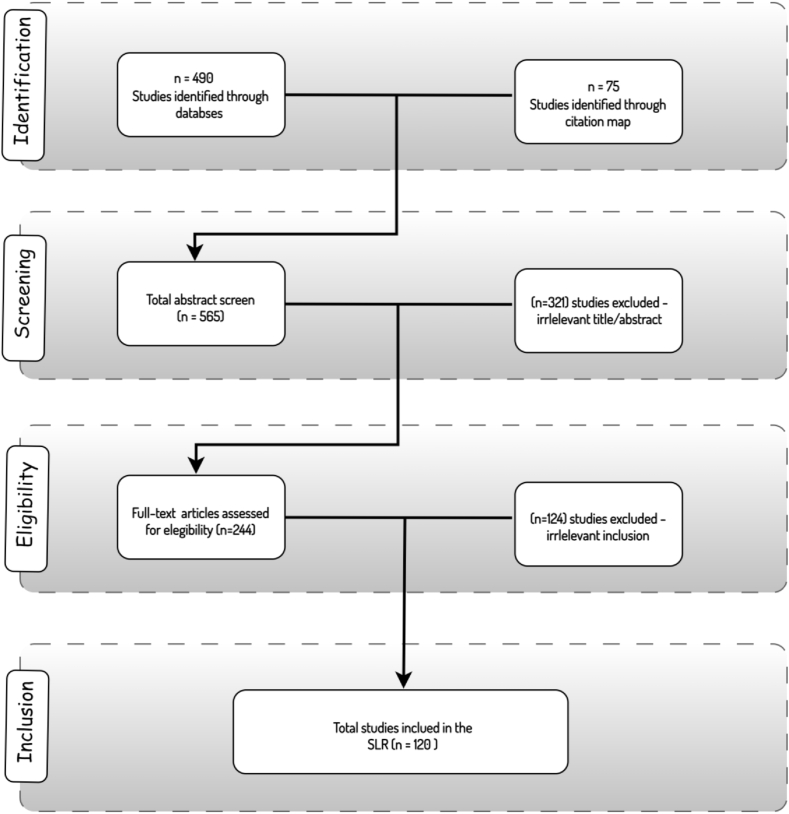
The paper selection strategy for the conducted SLR (using PRISMA model).

Fig. 1 illustrates the selection process based on the PRISMA framework. Initially, approximately 490 articles were identified based on the conducted keyword-based search. To augment this dataset, an additional 75 articles were included by reviewing the citations and references of the collected papers. This preliminary phase yielded a total of 565 records. Subsequently, a screening stage was carried out to eliminate any redundant or irrelevant articles. Both the title and abstract of each paper were scrutinized to ensure alignment with the inclusion criteria. Consequently, 321 records were excluded at this stage. Notably, some articles focused on KG embeddings applied to existing KGs, rather than the construction and application of educational KGs. Others discussed KG construction in domains unrelated to education but mentioned education as an illustrative application of KGs for industrial purposes. Following the screening phase, an eligibility assessment was performed by thoroughly examining the full texts of the remaining papers. This step led to excluding 124 records that did not meet the defined criteria. In the final stage of the SLR, 120 papers were identified as meeting the qualifications for inclusion in this comprehensive review of KG construction in the educational context.

**Fig. 2:**
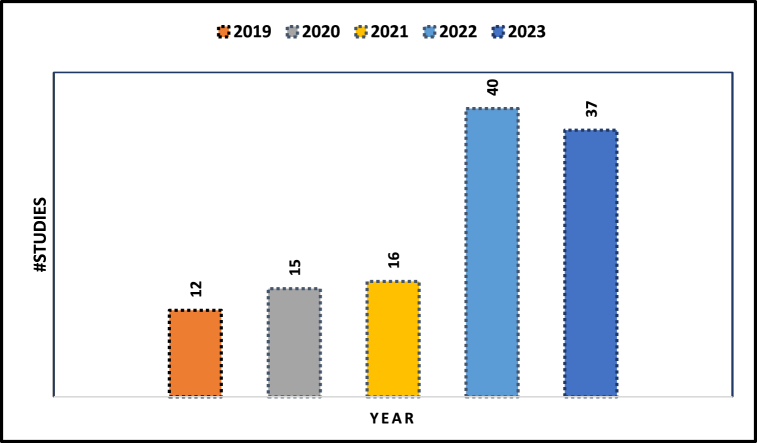
#Studies in the past five years that are included in the SLR.

Fig. 2 demonstrates the distribution of selected articles used in the conducted SLR. The figure shows a noticeable trend of increasing interest in utilizing KG for education. This is evident by the rising number of articles being published each year within the chosen timeframe. This implies that the education domain is experiencing a growing emphasis on KGs as a valuable tool or technology for various purposes, including curriculum design, personalised learning, semantic search, and other areas explored in this study. The following section provides a background of the key concepts relevant to KG and its application to education.

## 3 Domain-specific knowledge graph: concept and significance

In the realm of information organization and representation, KGs have evolved to offer a specialized approach known as domain-specific KGs. These domain-focused KGs cater to distinct subject areas or fields, providing a tailored means of capturing, structuring, and navigating intricate knowledge landscapes [9]. The significance of domain-specific KGs is rooted in their ability to provide in-depth insights and context within a specialized subject area. These KGs enable researchers, experts, and enthusiasts to navigate complex knowledge domains with precision and relevance. By capturing domain-specific relationships, KGs empower users to discover connections, trends, and patterns that might not be immediately apparent through traditional information retrieval methods. One of the primary advantages of domain-specific KGs is their capacity to foster a deeper understanding of specialized subjects [23]. These KGs allow users to explore intricate webs of information, grasping the nuances and interdependencies that define the domain. This, in turn, facilitates more informed decision-making, innovation, and discovery within specific fields [24]. Moreover, domain-specific KGs have practical implications across various sectors. In industries like healthcare, a domain-specific KG could map medical conditions, treatments, and patient data, offering comprehensive insights for medical professionals [5]. In finance, a KG might interconnect economic indicators, market trends, and financial instruments, aiding analysts in making informed predictions [[25], [26], [27], [28]]. More discussion of the semantic representation and relationships, as well as the relevant of KGs to education, is discussed in the following sections.

### 3.1 Semantic representation and relationships

To enable complex knowledge and context, semantic representation and linkages are crucial. Semantic representation is the process of embedding relationships and meaning into data so that computers can understand information's true importance rather than merely its syntax [29]. This strategy is critical in sectors where context and relationships are essential, such as KGs. Relationships are at the heart of semantic representation – the intricate connections defining how entities and concepts relate. In KGs, these relationships are not mere associations; they carry explicit meanings. For instance, in a biological KG, the relationship "is a part of" can signify the structural connection between an organ and an organism. This semantic understanding allows KGs to go beyond keyword-based searches and enable more profound insights.

Semantic relationships in KGs enable the creation of an interconnected knowledge ecosystem. Concepts are linked not just by superficial links but by relationships that capture the essence of how they interact. For instance, a literature KG might show the relationship "authored by" connecting authors to their written works. Such links empower KGs to provide a multidimensional view of information, which is crucial in contexts where understanding intricate connections is paramount. Furthermore, semantic representation and relationships are the building blocks for meaningful queries and analytics. With semantic data, queries can be more intuitive. Instead of asking for isolated facts, users can inquire about complex relationships. For instance, a query in a movie KG might seek all actors who starred in films directed by a specific filmmaker during a particular decade. This level of specificity demonstrates the power of semantic representation in catering to precise information needs.

### 3.2 Relevance of knowledge graphs to education

In the landscape of education, the emergence of KGs has brought forth a transformative wave, revolutionizing how knowledge is organized, accessed, and applied. The relevance of KGs to education lies in their capacity to overcome the limitations of traditional learning resources and offer a dynamic, interconnected, and personalised learning experience [30]. At its core, education revolves around the dissemination of knowledge and fostering a deep understanding of concepts. KGs align seamlessly with this purpose by providing a structured framework that captures the relationships between various concepts, topics, and entities. By interlinking these elements, KGs enable learners to traverse a web of knowledge, exploring subjects in depth and understanding how different pieces fit together.

One of the critical applications of KGs in education is personalised learning [31]. Every learner is unique, with distinct preferences, strengths, and gaps in understanding. KGs harness this diversity by tailoring learning pathways based on individual progress and needs. Through semantic representation and intelligent analytics, KGs identify optimal learning sequences, recommend relevant resources, and adapt the learning experience to each learner's pace and style. Moreover, KGs empower educators to craft enriched learning environments. Teachers can leverage KGs to design curriculum plans that align with learning objectives and map out the progression of topics. KGs provide insights into the relationships between concepts, helping educators anticipate potential challenges in understanding and curating targeted interventions [32].

**Fig. 3:**
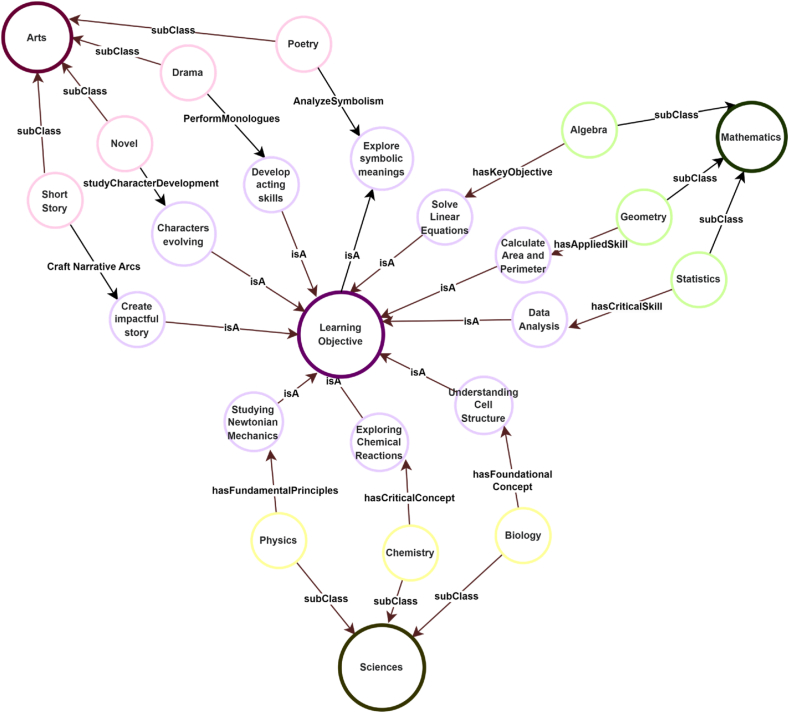
An example of a simplified representation of how different elements within a curriculum can be interconnected.

Learning analytics, another cornerstone of the educational landscape, thrives within the realm of KGs. These graphs capture intricate patterns of learner behaviour and progress, offering a comprehensive view of strengths and weaknesses. Analytics derived from KGs can inform educators about the effectiveness of teaching strategies, the impact of resources, and the overall trajectory of learning outcomes [9]. Institutions and educational platforms benefit from KGs as well. These graphs enable seamless content discovery, aiding learners in finding resources that resonate with their learning goals. Educational institutions can use the KG to map learning objectives to subjects, topics, and grade levels, thereby identifying gaps in their curriculum and making informed decisions to fill those gaps. Fig. 3 shows an example of a simplified representation of how different elements within a curriculum can be interconnected. In reality, curricula are much more detailed and intricate.

## 4 applications of knowledge graphs construction in education

**Fig. 4:**
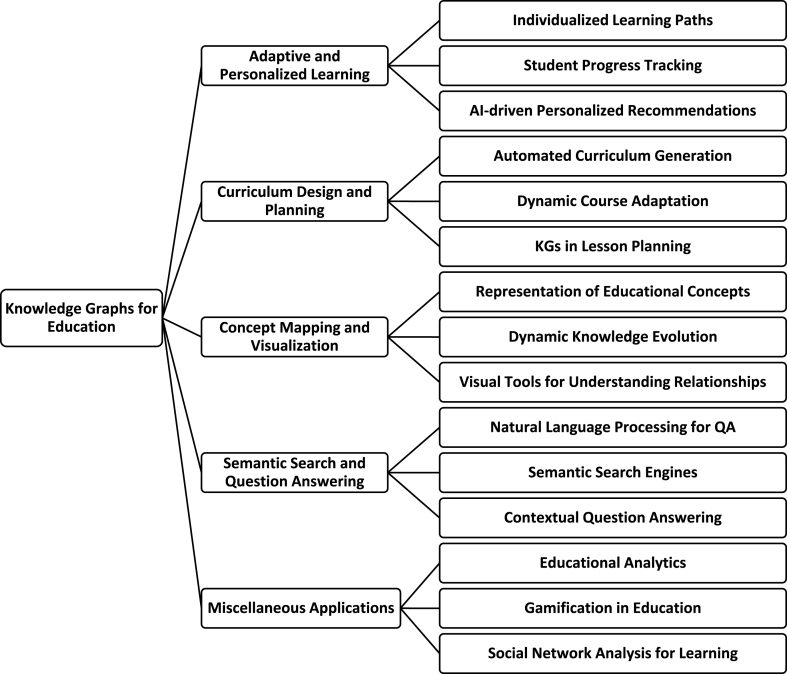
A taxonomy of the main applications of KGs in Education.

In this section, we navigate a vast body of academic work that harnesses the potential of Knowledge Graphs (KGs) to advance education. We will highlight significant achievements and acknowledge the challenges encountered during this journey. Our exploration centres on five core domains: Adaptive and Personalised Learning, Curriculum Design and Planning, Concept Mapping and Visualization, Semantic Search and Questioning Answering, and other pertinent miscellaneous applications. These domains serve as our guiding compass through the diverse and enriching landscape of educational KG applications. Within each domain, KGs hold the promise of delivering tailored, data-driven, and adaptive educational experiences. Throughout this examination, we meticulously scrutinize the methodologies employed, offering concise summaries for each approach. These summaries shed light on the specific functionalities of the constructed KGs, the techniques applied for knowledge extraction and construction, the characteristics of the knowledge bases, the necessary resources for KG development, relevant statistics about the KGs, the criteria used for evaluating KG construction methods, as well as their respective limitations and shortcomings. A taxonomy of the main applications of KGs in Education is illustrated in Fig. 4.

### 4.1 Adaptive and personalised learning

Personalised learning is an educational approach that aims to customize learning for each student's strengths, needs, skills, and interests. Each student gets a learning plan that's based on what they know and how they learn best. KGs can play an important role in achieving personalised learning in education. By representing and organizing information about learners and their progress, KGs can facilitate the adaptation of instruction to individual needs and characteristics. KGs are specially organized to present entities from any educational area and the relations between these entities. They can be used to represent and organize information about learners, their characteristics, and their progress, allowing for more effective adaptation of instruction. In this context, authors of [33] outlined the problem of personalised learning resource recommendation in the context of online education, discussed the importance of KGs, presented an algorithm for personalised recommendation, and described the construction of a personalised learning resource recommendation system. Lu et al. [34] introduced "RadarMath," an innovative, intelligent tutoring system catering to personalised math education. It offers automatic grading and customized learning support by implementing two grading models: DL-based for text-answer and STACK-based for formula-answer questions. Zhang et al. [35] proposed an adaptive learning method utilizing KG to personalise education. It involves multi-dimensional KG frameworks, attention mechanism-based classification, and navigation path generation with activation theory.

The importance of technology-supported education and cooperative learning and emphasizing the need for a recommendation system to support lifelong learning are discussed in the literature. For example, Xue et al. [36] introduced advanced methods for clustering students and recommending courses based on semantic and statistical dimensions. The study's experiments confirm the proposed strategy's practical applicability and efficiency in assembling student profiles and suggesting courses for various education levels. However, the discussion in this paper is limited in terms of data quality, scalability, biases in recommendations, and challenges in accurately capturing students' preferences and learning behaviours. Another study proposed a learning resource recommendation algorithm that combines a knowledge graph and an interest diffusion mechanism [37]. The author aimed to address the issue of information overload and assist learners in finding relevant resources. Another work focused on developing a personalised learning path recommendation system based on a cognitive graph [38]. The cognitive graph integrates KG and cognitive reasoning to visualize students' cognitive maps, allowing them to monitor their existing knowledge structures and cognitive states.

Adaptive education refers to an educational approach that tailors instruction and learning experiences to the individual needs, preferences, and progress of each student. In this context, Bai et al. [39] demonstrated the application of a variable incremental adaptive learning model based on a KG for online learning systems. This model aims to address challenges such as cognitive overload and confusion in online learning by utilizing a Kg and an improved FP-growth algorithm for data mining. Another study attempted to design an adaptive E-learning solution for adult learners in open education, addressing the challenges posed by diverse learner backgrounds [40]. The authors proposed solution involves constructing learner portraits based on various learner features and organizing learning resources using a KG. Incorporating KG technology for adaptive education, personalised learning, and education recommendations were also discussed and conveyed in Refs. [[41], [42], [43], [44], [45], [46], [47]].

The combination of KGs and machine learning techniques improves the prediction of students' academic performance and enables the identification of students at risk of course failure. In this context, Albreiki et al. [48] applied KGs and ML to assess and predict students' academic performance, identify students at risk of failing a course, and provide personalised interventions. Another study demonstrated that Deep Neural Network Architecture (DNNA) is effective when integrated with the KG to provide interpretable early warning recommendations for interactive learning processes [49]. The specific purpose of the KG is to represent the relationships and entities related to learning behaviour in an online learning platform. It is used to enhance the effectiveness of early warning recommendations. Another study discussed a hybrid reasoning approach of KG based on Reinforcement Learning - Multi Relational GCN reasoning integrated with reinforcement learning (RL-URGCN) [50]. The integration of ML and KG technologies to benefit the education domain was also examined and reported in Refs. [[51], [52], [53], [54], [55], [56], [57]]. Table 1 demonstrates a summary of selected KG construction approaches for adaptive and personalised learning.

**Table 1:** A summary of selected KG construction approaches for adaptive and personalised learning.

Ref.	KG Specific Purpose	Construction Algorithm(s)	Type of KB	KG Resource(s)	#Entites (e)/#Relations (r)	Evaluation Criteria	Limitation(s)
[58]	Learning assessment and recommendation	Bootstrapping construction strategy and BERT-BiLSTM-CRF	Schema-free	Subject teaching resources, Baidu Encyclopedia, and DBPedia	#e: 2202 #r: 3122	P, R, F1 measure, and case study	• Restricted presentation on the benefit of the constructed KG for student learning,
[33]	Learning Resource Recommendation	Ontology construction, Weighted Fusion Method	Schema-based	Learning resources	N/A	F1-score comparison, Efficiency analysis	•Limited focus on discrete mathematics, •Assumed optimal alpha, within a specific range, •Small-scale dataset
[34]	Intelligent Tutoring System for Math Education	DL-based Grading Model, STACK-based Grading Model,	N/A	Learning resources, Learner profiles, Grading models, Instructional concepts, Knowledge states, Learning interactions	N/A	Quadratic weighted kappa value, F1-score, Accuracy of grading models	•Limited focus on discrete mathematics, •Third-party reliance on STACK, •Small-scale initial deployment.
[35]	personalised learning path recommendation	multi-dimensional KG frameworks, attention mechanisms, and activation theory for path generation	N/A	Educational resources	N/A	Accuracy, effectiveness, and quality of adaptive learning services.	•Refining frameworks for various learning scenarios, •Scalability in path generation and • Addressing limitations in automatic cognitive perception within the KG.
[36]	Students' clustering and course recommendation	knowledge network, machine-learning	Schema-free	Student profiles, course data, and features extracted from textual data	#e: 675 #r: 1033	P, R, Accuracy, F1_Score RMSE and MAP	•Data quality, •Scalability, •Biases in recommendations, •Challenges in accurately capturing students' preferences and learning behaviours.
[37]	learning resources and guiding recommendations	N/A	Hybrid-based	Educational content	N/A	N/A	•Lack of discussion on KG construction. •Lack of discussion on the evaluation criteria
[38]	Visual representation of learning paths	Concept maps	Hybrid-based	knowledge units	N/A	Case Study	•Challenges related to the accuracy of cognitive reasoning, •Lack of rigorous evaluation metrics.
[39]	Adaptive learning experiences for students	An improved version of the FP-growth algorithm	Schema-based	Students' searches within the online learning system	N/A	Students' satisfaction via surveys	•Poor evaluation metrics, •Limited discussion on the collected entities and relationships.
[40]	Adaptive E-learning for Adult Learners in Open Education	Manual extraction of entities and relationship	Schema-based	Learning resources of the course “Principle and Application of Database System”	N/A	Subjective evaluation of 30 learners who participated in an online course.	•Potential difficulties in managing and updating the knowledge graph as the course content evolves. •Limited discussion on mechanisms used to construct the KG as well as the size of the resultant graph.
[48]	Identify students at risk of failing a course and provide personalised interventions.	Ontology mapping	Hybrid	Courses offered by the College of Information Technology at UAEU between 2016 and 2021.	N/A	P, R, F1-score, and Accuracy	•Limited dataset size, •Lack of statistics with regards to the size of resultant KG, •Lack of subjective evaluation and application of downstream task.
[49]	Development of an interpretable early warning recommendation mechanism for learning behavior.	DNNs	Hybrid	AI-enabled online learning platform	#e: 1204	AUC, RI, F1 score, and Multi-task Learning Gain (MTL-Gain).	•There are in-depth logical designs and topology verifications for concept classes of learning content, •A need to improve the dependability of feedback and early warning accuracy.

### 4.2 Curriculum design and planning

Curriculum design and planning encompass the systematic development of educational programs, courses, and learning experiences. It involves a thoughtful alignment of learning objectives, content, instructional methods, assessment strategies, and resources to foster meaningful learning among students [59]. Incorporating KGs into curriculum design and planning offers a data-driven, learner-centric approach that enhances the quality, relevance, and effectiveness of educational offerings [60,61]. For example, Yang et al. [62] constructed a KG to assist water conservancy students in understanding the relationships between courses, knowledge units, and terminology. It was designed to provide a structured representation of water conservancy education data for efficient learning and knowledge retrieval. Authors of [63] developed an ontology called EducOnto and a KG called EduKG to assist in university curriculum recommendation. EducOnto models the period between high school and university and contains concepts like student, curriculum, major, specialty, etc. Zablith [64] designed a KG, the aim of which is to establish semantic links between social media content and formal course entities, enabling students to integrate and access transdisciplinary social media content within formal courses.

A new framework called ModelsKG for constructing a multimodal curriculum knowledge graph that integrates PaddleOCR and DeepKE was developed [65]. The aim of this framework was to enhance intelligent education by linking and reorganizing multi-modal knowledge, enabling intelligent search, link construction, quantitative analysis, and intelligent recommendation. Another methodology is proposed to facilitate the dynamic generation of a STEAM (Science, Technology, Engineering, Arts, and Mathematics) KG that supports interdisciplinary instructional design [61]. In particular, the study aims to enable teachers to design appropriate learning themes that align with curriculum standards. Authors of [66] developed three KGs, namely Course KG, Teacher KG, and Course topology KG. These KGs were constructed to facilitate course-teacher matching, optimize course offerings, and ensure curriculum coherence. Another study constructed an ontology-based KG for the Management Principles course in the Management Information System (MIS) curriculum to enhance teaching effectiveness and enable personalised content delivery in modern education [67]. Yao [68] discussed the construction and application of a multi-modal KG for blended teaching. The author's word emphasized the integration of various forms of resources like pictures, audio, and video into the KG to enhance teaching and learning experiences. Gao et al. [69] attempted to visually display relationships between different components of the curriculum, such as chapters, sections, and knowledge points, using KG technology. Visualizing the KG vividly was discussed in Ref. [70]. It produces the KG of database micro-lectures based on Neo4j and designs various system functions according to several roles.

KGs are also constructed and used to optimize the process of teaching. For example, Hu et al. [71] developed an Internet Fraud Knowledge Graph (IFKG) to organize correlations between internet fraud cases and relevant knowledge points within the cases, facilitating more effective and personalised teaching. Another study focused on the development of an automated and personalised assignment system for programming tasks in an educational context [72]. In this study, the authors developed a system that takes into account task complexity, learner's programming errors, and knowledge level to provide tailored programming assignments to students. Another study proposed Educational Knowledge Graph 2.0 [73] that integrated big data and deep learning techniques to construct and optimize a more intelligent and effective KG for education. This research used the C language programming course as experimental data to quantitatively validate the model's efficacy. The approach has demonstrated great improvement in teaching quality and enables "multi-directional adaptation" among instructors, courses, and students. Developing a KG to provide feedback to learners engaged in collaborative online learning activities was proposed [74]. The specific purpose of this study is to automatically transform group discussions into KGs to characterize group understanding and provide real-time automated feedback to student groups. Additionally, it supports group comparison based on graph algorithms. The same direction of research was also applied in Refs. [75,76]. Curriculum design and planning are key concepts in education, whereby KG technology has demonstrated success in various other applications [[77], [78], [79], [80], [81], [82]]. Table 2 shows a summary of selected KG construction approaches for curriculum design and planning.

**Table 2:** A summary of selected KG construction approaches for curriculum design and planning.

Ref.	KG Specific Purpose	Construction Algorithm(s)	Type of KB	KG Resource(s)	#Entites (e)/#Relations (r)	Evaluation Criteria	Limitation(s)
[62]	Improve curriculum and assist students' learning and understanding.	Water Disciplines Entity-Relationship Joint Extraction (WDERJE) framework	Schema-based	Water conservancy educational big data	#e: NA #r: 180,000	F0.5-score	•Limitations in handling evolving or dynamic knowledge.
[63]	University curriculum design	Manual	Schema-based	Data collected from French students through surveys	#e: 5452 #r: 27	task-based evaluation	•Limited discussion on the construction algorithm. •Poor evaluation techniques,
[64]	integrating social media contents in formal courses	Semantic linking techniques	Hybrid	formal course entities, social media materials	#e: 230#r: N/A	Case study	•The experiment is being conducted within a specific course in the IS domain, which could introduce biases. •The study did not analyze professors' perspectives, which could provide valuable insights. •Some of the social features developed in the tools are not being utilized fully. •The potential for differences in concept labelling by different students in crowdsourced ontology concepts. •Lack of analysis on the implications of dynamically constructed program goals and learning objectives.
[65]	Design a structured representation of curriculum knowledge using multimodal KG.	DeepKE and PaddleOCR	Hybrid	Course materials, teaching videos, and speech content	N/A	Case study	•The DeepKE model's generalization ability might be limited due to the relatively small labelled training data. •The paper doesn't elaborate on the challenges of multimodal fusion, •Lack of quantitative results or analyses of the framework's performance.
[61]	Supporting STEAM learning theme design	BERT and tensor decomposition	Schema-free	Encyclopedia, OpenKG, and national discipline curriculum standards	N/A	P, R, F1, MRR, and Hits@N	•Further research is needed to address the interpretability mechanism of KGs for STEAM interdisciplinary semantic learning.
[66]	Curriculum system improvement for higher education	Various algorithms are used to construct three KGs	Schema-free	syllabuses of courses and external data sources	N/A	Subjective criteria such as positive feedback from teachers and students through surveys.	•The effectiveness of the approach relies on the availability and accuracy of course syllabuses, teacher resumes, and related data. •The approach might require customization for different educational contexts.
[67]	Enhancing MIS course curriculum.	Top-down ontology modeling that maps the ontology model into a KG.	Schema-based	Course material	N/A	Case study	•Limitations in its generalizability to other domains. •Incomplete or inaccurate ontology definitions could lead to limitations in the KG's quality.
[68]	Supporting blended learning	Association mining	Hybrid	Textbooks, curriculum standards, etc.	N/A	Case study	•Lack of adequate description of mechanisms used in constructing and evaluating the KG.
[69]	A visual representation of the curriculum including chapters, sections, and knowledge points.	Vocabulary mining, entity recognition, and knowledge extraction.	Schema-free	High school information technology textbooks	N/A	Case Study	•Potential errors in knowledge extraction, •No discussion on the algorithms used for constructing the graph.
[71]	Optimizing the teaching of internet fraud cases.	Ontology mapping and relational mapping	Schema-based	Internet fraud cases, their characteristics, victims, platforms, fraud processes, technologies, losses, and prevention methods.	N/A	Case study (improvement in students' understanding and awareness of internet fraud)	•Limitations related to the size of the fraud knowledge graph and the potential absence of certain individual cases. •The KGCT model is under development, thus ongoing refinement and improvement are needed.
[72]	Education technology and learning analytics	ontology-based learner modeling techniques	Schema-based	Programming records from an online programming platform	N/A	quasi-experiment, standardized tests, and experimental group	•The study's findings are based on a relatively small sample size of 38 participants, •Lack of technical details of how these graphs are utilized or how the, •The study does not explore the potential impact of KGs on group students or facilitate peer assistance.

### 4.3 Concept mapping and visualization

The synergistic integration of concept mapping, visualization techniques, and KGs has garnered significant attention for its potential to revolutionize the learning experience. Concept mapping, rooted in cognitive theories of learning, empowers students to visually organize complex information, thus fostering deeper understanding and retention [83]. When paired with visualization techniques, which offer a means to graphically represent intricate relationships, the potential for enhancing cognitive engagement becomes even more pronounced [84,85]. The visual-spatial representations provided by concept maps and visualizations not only aid in breaking down intricate concepts but also facilitate extracting meaningful insights from interconnected information [30]. Various studies have examined the interplay between concept mapping, visualization techniques, and KGs, and their collective impact on student learning outcomes. For example, Li et al. [86] developed a multi-source education KG for college curricula in the major of Electronic Information. The goal is to enhance learning efficiency by providing a comprehensive understanding of relationships between different concepts and courses using visualization techniques. In the same direction, Su et al. [58] aimed to represent the interconnected knowledge points within educational subjects, enabling non-linear teaching approaches, microteaching, learning assessment, learning navigation, and personalised resource recommendations. For the benefit of students taking programming classes, a KG on the subject of Python is constructed in Ref. [87]. The experimental findings reported in the paper demonstrated that the concepts are described more clearly and that the logical linkages between the knowledge points are deduced based on the students' questions after three applications on the constructed KG. Building KGs for the aid in the institution and dissemination of educational content has also been discussed in Ref. [88]. The integrated KG aimed to provide a framework for connecting historical events, literature, geography, and other related concepts to facilitate interdisciplinary learning. Integrating heterogeneous data sources into a unified KG has also been reported in Ref. [89], whereby the authors developed EDUKG; a comprehensive educational KG that represents knowledge topics, educational resources (including teaching materials and exercises), and external heterogeneous data sources related to K-12 education.

KG visualization in education is a powerful approach that enhances comprehension, exploration, and engagement with complex educational content. For example, Tang et al. [4] constructed a KG to visually represent the curriculum content system, integrate online learning resources to establish matching relationships between knowledge points and resources and help students quickly locate and understand the relationships between knowledge points. In Ref. [90], the authors constructed a KG from unstructured course materials to facilitate learning in the field of cybersecurity education. The article presents a bottom-up approach for identifying key entities and relations, leading to the development of an ontology framework that can be used to build KGs. The knowledge graphs serve two main purposes: concept visualization and question answering. They are designed to visually represent complex concepts in cybersecurity, aiding students in understanding and implementing project challenges. Additionally, a chatbot is developed to answer student queries related to lab setup, concepts, and projects. Authors of [91] developed IE-DEKG model which utilized various algorithms for different modules, including mutual information, adjacent information entropy, topic modelling, association rule mining, and pattern matching for extracting instructional concepts and identifying educational relations. IE-DEKG mode seeks to dynamically construct educational knowledge graphs using instructional concept extraction and educational relation identification techniques. The model shows promising precision, recall, and F1-score results. Another study demonstrated the application of a visual KG in teaching Assembly Language Programming and how this technology can enhance teaching effectiveness and student engagement [92]. Visualization of KGs and how they can be used to support education have also been discussed in Refs. [[93], [94], [95], [96], [97]]. Further, KG construction to benefit concept mapping in education was also elaborated in Refs. [60,[98], [99], [100]]. Table 3 summarises selected KG construction approaches for concept mapping and visualization.

**Table 3:** A summary of selected KG construction approaches for concept mapping and visualization.

Ref.	KG Specific Purpose	Construction Algorithm(s)	Type of KB	KG Resource(s)	#Entites (e)/#Relations (r)	Evaluation Criteria	Limitation(s)
[86]	Improve learning outcomes	Ontology based concept mapping	Schema-based	Textbooks, course slides, and course syllabi	#e: 60,000 #r: 80,000	Correlation analysis, ranking entities	•Limited discussion on the KG construction algorithms. •Poor evaluation methodology.
[4]	Visual representation of the curriculum contents.	N/A	Schema-based	Chinese University MOOC, PTA, and Rain Classroom.	N/A	Average scoring rates	•Focus on K12 education disciplines, with less research in higher education disciplines. •Lack of comprehensive research on the combination of theory and practice in higher education. •Limited discussion on the KG construction and evaluation.
[90]	Visual representation of complex concepts in cybersecurity	NER and ontology mapping	Hybrid	Lecture notes, project lab manuals, quizzes, etc.	#e: 62 #r: 44	Surveys and interviews	•Potential difficulty of accurately extracting entities and relations from highly varied and complex unstructured texts. •The approach was not validated on downstream tasks.
[58]	Identifying subject teaching resources	BERT-BiLSTM-CRF	Hybrid	Teaching resources (syllabuses, textbooks, lesson plans) and internet encyclopedia texts (similar to DBpedia for expansion).	#e: 1225 #r: 1722	PMI, NGD, baselines comparison	•Inadequacy in capturing all knowledge points, •The choice of parameters such as context window size (k) and threshold values affects the graph's structure, •Potential limitations in the coverage of internet encyclopedia texts.
[91]	Knowledge building community	Mutual information, adjacent information entropy, topic modeling, association rule mining, and pattern matching	Schema-free	Students' notes, reflections, and summarizations in the field of physics subjects.	The paper does not explicitly mention the exact numbers, but it states that a total of 7339 junior middle school physics texts were collected for the dataset.	precision, recall, F1-score, and qualitative analysis	•Sensitivity to parameter settings, •Generalizability to other domains, •Scalability to larger datasets, •The challenge of ensuring high-quality educational relations through automated techniques.
[101]	Visualizing and querying medical knowledge.	Determining entities, attributes, and relationships based on user demand analysis and data sources.	Schema-based	Internet medical encyclopedia, medical encyclopedia, and other medical knowledge sources.	N/A	Clarity, accessibility, and comprehensiveness of the visualized knowledge map,	•No proper discussion on the mechanisms followed to construct the KG, •Poor evaluation techniques.
[102]	The interactive dictionary that offers descriptions, meanings, and semantic networks of programming skills	Rule-based concept mapping	schema-based	Wikipedia	N/A	A comparison of PS-Dict with other computer dictionaries.	•Lack of discussion of the accuracy of the heuristic rules used for retrieving Wikipedia articles, •No proper discussion of the mechanisms followed to construct the KG.
[88]	Liberal arts subjects	"four-step method" involving domain experts, semantic annotation, and data enrichment.	Schema-based	historical events, literary works, geographical information, and related educational content,	N/A	N/A	•The article lacks specific data or statistics regarding the size and complexity of the KGs created. •It does not explore the technical details of constructing the KG, making it challenging to replicate the process. •The integration of the KG relies on manual annotation and may benefit from more automated methods. •Practical applications and benefits for educators and students are not elaborated upon in detail.
[89]	Educational KG Construction and Management	NLP and EduLink	Hybrid	LRMI Standard for educational resources, and external data sources, such as schema.org, YAGO, Wikidata, and diverse online data.	#e:>2.5 M	A comparison with other existing educational KGs using data sufficiency metric	•Lack of specific details on construction algorithms. •The challenge of indexing and linking heterogeneous online data effectively. •The complexity of representing rhetorical roles in educational knowledge.
[103]	Internal policy control conceptualization and visualization in higher education	Adhoc		CNKI database		mean Silhouette	•Limited data sources, •limited application scope, •poor evaluation metrics, •KG embedding was not properly demonstrated and evaluated

### 4.4 Semantic search, QA, and recommender systems

KGs have significantly transformed semantic search and question-answering (QA) in the realm of education. Leveraging their interconnected structure and semantic richness, KGs enhance search results and answers' precision, contextuality, and depth. Therefore, various efforts have been made to benefit from KG technology, offering contextual and relevant information for teachers and students. In this context, Fang et al. [17] proposed BGNN-TT model improves multi-hop reasoning in educational KGs by utilizing a bilinear graph neural network and a two-teacher knowledge distillation approach. Nguyen et al. [104] integrated an ontology-based Rela-Ops model and a KG (Rela-KG model) so as to offer a practical approach to organizing and querying educational content for the Fundamentals of Database Systems course. The system serves as a foundation for an intelligent querying system that assists students in searching, comparing, and retrieving relevant course knowledge. An intelligent QA system based on KG is proposed in Ref. [105]. The key purpose of this KG is to support the intelligent QA system for science and technology intermediary services. It serves as the backbone for knowledge storage and retrieval, allowing the system to understand user queries and provide relevant answers based on structured data. Authors of [106] designed a KG to facilitate intelligent question-answering and feedback to students and teachers. This system enables knowledge modelling, accurate knowledge acquisition, and better teaching feedback by integrating KG, smart Q&A, and big data technologies. In a prototype conducted by Ref. [107], the authors demonstrated a need for a KG to develop a QA system for remote schools.

Authors of [108] presented MOOC-KG as a solution to improve online learning resource utilization by collecting and organizing information from various MOOCs, platforms, universities, teachers, and courses. In Ref. [109], the authors presented a four-step approach using semantic web technologies to identify and evaluate prerequisite relationships between concepts accurately. Authors of [110] developed an intelligent course content recommendation system based on a multi-view KG to facilitate real-time learning and instructional support. The system uses a multi-modal approach, incorporating information from student learning, searching activity patterns and course-specific content. Authors of [111] developed the first intelligent question-answering bot on Chinese-based MOOCs (named Xiao-Shih). Xiao-Shih features a built-in system for self-enrichment that allows the knowledge base to be expanded through public, community-based question responding. Another study reported a novel three-stage framework to remotely supervise the extraction of course concepts from MOOCs across various domains to reduce the labour-intensive task of human annotations [112]. Developing and integrating KGs to benefit MOOCs have also been elaborated in Refs. [2,3,113,114]. Further, integrating KG technology to benefit the semantic search and QA systems was discussed and reported in Refs. [115,116]. Table 4 summarises selected KG construction approaches for semantic search and QA applications.

**Table 4:** A summary of selected KG construction approaches for semantic search and QA.

Ref.	KG Specific Purpose	Construction Algorithm(s)	Type of KB	KG Resource(s)	#Entites (e)/#Relations (r)	Evaluation Criteria	Limitation(s)
[17]	Educational question-answering in the context of sustainable urban living	semantic parsing-based methods and retrieval-based methods	Hybrid	MOOCCube KG	52,195 triples	Hits@1, Hits@3, Hits@5, and MRR	•Lack of discussion on the effectiveness of the two-teacher knowledge distillation process. •Potential challenges in handling complex reasoning scenarios.
[104]	Intelligent querying system	Rela-Ops model to define concepts, relations, operators, and rules,	Schema-based	Content of the Fundamentals of Database Systems course	N/A	Query accuracy, user satisfaction, and the system's ability to recommend related knowledge.	•Potential limitations in scalability of the system to handle diverse queries. •Poor discussion of the mechanisms used to construct the KG as well as certain resultant statistics.
[109]	Searching for course prerequisites	Matching with DBpedia	Schema-based	DBpedia	N/A	precision, true positives, and false positives. The paper compares the results across different domains using various search and pruning strategies.	•The lack of annotated prerequisite datasets in domains beyond those used for training limits the generalizability of the supervised model. •The use of Common Memberships as a search strategy might not be suitable when categories are far from the main domain or have very few members.
[105]	Intelligent question-answering system for science and technology intermediary services	jieba Chinese word segmentation tool and the Aho-Corasick algorithm	Hybrid	Structured data provided by collaboration unit	5 types of entities, 15 attributes, and 6 semantic relations	Visualization	•Limited data size •There is a need to develop a tool for automatically adding technical terms to a custom dictionary. •There is a need to optimize the framework and algorithms for broader coverage.
[106]	Building a structured semantic knowledge base for high school course content	Reverse Maximum Matching (RMM) and Conditional Random Fields (CRF)	Hybrid	high school course materials,	N/A	N/A	•The paper lacks specific technical details about the construction of the knowledge graph and its internal structure. •Also, the paper lacks details on the evaluation criteria used to measure the performance of the system.
[110]	Real-time learning and instructional support	multi-modal action fusion module	Hybrid	Course materials (lecture slides, notes, videos), student learning activity data, discussion forum posts, and DBpedia	N/A	P, NDCG, F1-score, explainability score	•No quantitative figures regarding the size and complexity of the KG, •While the article discusses explainability, it doesn't provide in-depth insights into the explainability methods used.
[117]	Semantic Search and QA in the domain of Information Management Systems	NER-Bi-LSTM-CRF for Named Entity Recognition and RR-Bi-LSTM-CRF for Relationship Recognition	schema-based	MIS program at Beijing Jiaotong University	N/A	F1 Score and mAP (mean Average Precision)	•Further improvement is needed in answering recommendations since the system sometimes falls short of user expectations.
[60]	QA and course allocation scheduling	NLP for entity and relation extraction	Schema-based	Structural educational information system	N/A	Case study	•Poor assessment methods, •restricted KG resources, •limited scope
[107]	QA for unstructured educational text.	NLP-BERT	Hybrid	Extensive text sources.	N/A	N/A	•Poor discussion on mechanisms followed to construct the KG, •No evaluation criteria or proper case studies to measure the performance of the constructed KG.
[108]	Explore, search, and select online courses	Entity extraction (NER), relation extraction, and entity alignment	Schema-based	XuetangX, ICourse, Coursera, and EDX.	#e: 28,591	Statistical analysis of providers, subjects, languages, and course ratings.	•The article mentioned the periodic update and crowdsourcing for knowledge updating but did not elaborate on how the quality and credibility of contributed updates will be ensured. •the scalability and performance of the graph database (Neo4j) for larger-scale knowledge graphs could be a potential limitation not extensively addressed in the article.

### 4.5 Miscellaneous applications

The above sections discuss some of the KG applications in specific, well-defined and widely recognized categories; however, a realm of miscellaneous and innovative use cases continues to reshape the education industry and relevant sub domains in unexpected ways. This section delves into the intriguing and less-explored applications of KGs, highlighting their adaptability and transformative potential across diverse fields. For example, authors of [118] incorporated an "entity-event KG" approach for managing and structuring human resources management data within public administrations. The paper aims to demonstrate the application of this approach to the case of education personnel management. In Ref. [119], the authors demonstrated the use of the domain KG to enhance piano teaching by integrating various curriculum resources, presenting knowledge intuitively, and helping students learn more efficiently. The authors combined deep neural networks, including Convolutional Neural Networks (CNN) and Recurrent Neural Networks (RNN), to construct the multimodal knowledge Atlas. The construction of a domain KG for primary school mathematical operation literacy to assist in the assessment and improvement of students' mathematical skills was proposed in Ref. [120]. In this KG, the authors represented and organized primary school mathematical operation literacy knowledge, including knowledge dimensions, cognitive goals, and abilities.

KG technology, exceptionally an interdisciplinary KG based on an open domain, can significantly enhance the utilization, presentation, and recommendation of digital learning resources in the context of lifelong learning. In this context, Yu [121] created an ecological chain of supply for lifelong learning resource bases using Kg. This includes improving resource utilization, visual presentation, intelligent recommendation, question answering, and cross-resource association. Zheng et al. [1] contributed to this endeavour by developing an Automatic Activated and Unactivated KG (AAUKG), which serves as a tool to visualize, monitor, and facilitate collaborative knowledge building within the context of Computer-Supported Collaborative Learning (CSCL). Developing a power grid KG was reported by Ref. [122]. The authors designed this KG to serve as an intelligent education platform, enabling teachers, students, and parents to access, analyze, and understand power grid technology and its related educational resources. Automatic construction of a KG is also reported in Ref. [123]. The paper discussed a method for constructing a course ontology by combining automated data acquisition from the internet with manual annotation. This approach ensures the richness of knowledge in the course ontology.

Enhancing the integration of theoretical and practical knowledge in computer networking courses in secondary vocational schools was discussed in Ref. [124]. The paper used a bottom-up approach for KG construction. It involves data acquisition from sources such as e-textbooks, Zhihu, and w3cschool, keyword extraction using LDA, TF-IDF, and TextRank algorithms, relationship extraction using rule-based methods, and knowledge storage using the SmartKG tool. Investigating KGs for data-driven decision-making, resource aggregation, problem-solving, and adaptive learning systems has been reported in Ref. [125]. The authors constructed educational KGs through knowledge expression (primarily ontology-based), knowledge extraction (including concept, relationship, and attribute extraction), and knowledge visualization (representing complex knowledge visually). CKGG is a Chinese KG for the high school geography curriculum [126], this KG is designed as part of a long-term research project to improve students' computer-aided education. The authors transformed and integrated different types of geographic data from various sources, including gridded temperature data in NetCDF, precipitation data in HDF5, solar radiation data in AAIGrid, polygon data in GPKG, climate and ocean current data in images, and government data in tables, using GeoNames and Wikidata as a foundation. Accessed as Linked Data, the current edition of CKGG has 1.5 billion triples. Applying KG technology in education has been elaborated, discussed, and reported in various miscellaneous applications [60,68,127,128]. Table 5 summarises selected KG construction approaches for miscellaneous educational applications.

**Table 5:** A summary of selected KG construction approaches for miscellaneous applications.

Ref.	KG Specific Purpose	Construction Algorithm(s)	Type of KB	KG Resource(s)	#Entites (e)/#Relations (r)	Evaluation Criteria	Limitation(s)
[118]	Education in public administration	AllegroGraph	Schema-based	core vocabularies of the European Union	N/A	use-case scenario	•Implementing the model across various public administration contexts.
[119]	Enhance piano teaching	Deep neural networks	Hybrid	Encyclopedia websites and related piano websites containing text, pictures, and videos.	N/A	Accuracy, P, R, and F1-score	•Limited discussion on the size of the constructed KG, including a number of entities and relationships.
[121]	Creating an ecological chain of supply for lifelong learning resource bases	NLP tools for knowledge extraction, entity alignment, relation extraction, and rule reasoning.	Hybrid	Lifelong learning digital resource database, Baidu Encyclopedia, Wikipedia, and multimedia data sources	N/A	Case Study	•Poor evaluation metric, •Limited discussion on the knowledge extraction techniques, which restricts future efforts to reproduce the work.
[1]	Computer-Supported Collaborative Learning	BERT-BiLSTM-CFR	Hybrid	Online discussion transcripts from the CSCL environment.	N/A	various aspects of CSCL, including collaborative knowledge building, etc.	•Restricted sample size from one university. •The focus on a single collaborative learning task due to the COVID-19 pandemic
[122]	Understanding power grid technology and its related educational resources.	Concept mapping via NLP techniques	Hybrid	China National Knowledge Infrastructure (CNKI).	#e: >200 K	Case study	•Challenges in maintaining and updating the KG, •The complexity of accurately representing all facets of power grid technology within the graph.
[16]	Representation of MOOC resources across platforms	Word embedding-based approach to link concept mentions to Wikipedia entries	Schema-free	Coursera, EDX, XuetangX, and ICourse.	#e: 52,779 #triples: >300,000	Accuracy, user feedback, and comparison with existing KGs	•The accuracy of concept extraction may vary based on the quality of the text and available Wikipedia entries. •The accuracy of concept extraction may vary based on the quality of the text and available Wikipedia entries.
[124]	To enhance the integration of theoretical and practical knowledge in computer networking courses in secondary vocational schools	bottom-up approach, LDA, TF-IDF, and TextRank algorithms, rule-based methods	Hybrid	e-textbooks, Zhihu, and w3cschool.	#e: 239 #r: 521	Case study	•Poor evaluation metrics, •Potential limitations in scalability of the approach to larger datasets or different subject areas.
[120]	Primary school mathematical operation literacy	Concept mapping with the domain knowledge	Hybrid	Educational materials related to primary school mathematical operation literacy.	N/A	Case study	•Lacks details on the technical aspects of constructing the KG, •It also does not provide quantitative data on the size and complexity of the KG, •The paper does not discuss potential challenges or limitations in implementing the KG in real educational settings.
[125]	Educational Technology and Artificial Intelligence in Education	Primarily ontology-based knowledge extraction	Schema-based	Educational materials, textbooks, online courses, and other educational content.	N/A	N/A	•The article does not examine deeply the technical aspects of educational KG construction or provide concrete examples of specific educational KG. •lacks specific data or statistics regarding the size and complexity of these KGs, •It does not provide detailed solutions or insights into overcoming these limitations.
[129]	Solving high school mathematical exercises	Complex, Triangle, Conic and Solid	Schema-free	Crowdsourcing and domain experts.		Accuracy, P, R and F1 measure.	•Limited resources used for KG construction, •limited targeted audience
[130]	Link Prediction	Adhoc		Knowledge Forest, Wikipedia		Mean Rank and Hits@10	•Insufficient structural and literal embedding models were used

## 5 Summary of the state-of-the-art, discussion, and implications

The above sections demonstrate advancements that are driven by the increasing recognition of the key role that KGs play in enhancing personalised learning, curriculum design, concept mapping, and educational content recommendation systems. One notable advancement lies in the integration of semantic web technologies and natural language processing techniques to build rich and dynamic KGs. These KGs are no longer static repositories of information but are designed to capture the evolving and contextually relevant knowledge within the educational landscape. Additionally, there has been a surge in the utilization of machine learning algorithms for entity recognition, relation extraction, and concept linking, enabling the automatic population and refinement of KGs from vast textual resources. These advances not only contribute to the creation of more comprehensive and up-to-date educational KGs but also hold the promise of transforming how educators and learners’ access, interact with, and benefit from structured educational knowledge. However, this literature review exhibits certain limitations and inadequacies that are commonly observed in various works. These limitations can be summarized and aggregated as follows:

**Limited Discussion on Knowledge Extraction Techniques:** Knowledge extraction techniques embody methods and processes used to convert unstructured or semi-structured information into structured knowledge that can be stored, analyzed, and utilized in KGs. These techniques play a fundamental role in extracting educational content, relationships, and semantics from diverse data sources such as textbooks, research articles, lecture notes, and online resources. However, there are several limitations regarding the discussion and documentation of these techniques in the examined approaches. This opacity restricts the ability of other researchers to replicate or build upon the work, hindering progress in the field. Further, knowledge extraction in education often depends heavily on context. Different educational settings, subjects, and levels may require tailored approaches to extract relevant knowledge. However, several examined research papers have not sufficiently addressed these context-specific extraction techniques, making it challenging to adapt them to diverse educational scenarios. Tackling this limitation is crucial for advancing the field of educational KGs, as it requires a concerted effort to promote transparency, standardization, and the development of context-aware knowledge extraction methods that can better serve the multifaceted landscape of education.

**Lack of Standardization**: The absence of standardized formats or ontologies for educational KGs is another notable issue. Unlike more established KGs in domains like healthcare or finance [5,6,131], educational KGs lack a universally accepted structure. This makes sharing, integrating, or comparing KGs across different educational platforms or institutions challenging. The key problem of this inadequacy is the diversity of the educational ecosystem; the academic domain is incredibly diverse, ranging from K-12 education to higher education, vocational training, and lifelong learning. Each subdomain has specific educational objectives, curricula, and learning resources. Consequently, the structure and content of KGs can vary widely based on the educational context. Further, educational KGs draw data from a multitude of heterogeneous sources, including textbooks, research papers, course syllabi, learning management systems, and student records. Each of these sources may have its own data format, schema, and semantics. Standardising these diverse data sources into a single coherent KG format is a complex task. Addressing the lack of standardization in educational KGs requires collaborative efforts from academic institutions, standards organizations, and technology providers. Developing common ontologies, defining data interchange standards, and promoting best practices in data representation are essential steps towards achieving greater standardization. Additionally, ongoing efforts in the Linked Data community and initiatives like Schema.org are making strides in providing standardized schemas and metadata for educational resources.

**Limited Interoperability**: Many educational institutions and platforms have developed their KGs to suit their specific needs. However, these KGs often operate in isolation, limiting their interoperability with other systems. This lack of interoperability restricts the potential for creating a broader educational network that can harness KGs for a variety of applications. In fact, unlike some other domains where ontologies (formal representations of knowledge) have been widely adopted, the educational field lacks a universally accepted ontology or schema. This absence of common ontologies makes it challenging to ensure that data from various educational institutions or platforms can be seamlessly integrated into a unified KG. The limited interoperability is relevant to the lack of standardization. Without standardization, achieving interoperability between different educational systems and platforms becomes problematic. Interoperability enables the exchange of educational data, resources, and analytics across institutions and tools. It ensures that another can effectively utilize an educational KG created by one institution.

**Sparse and Incomplete Data**: Constructing a comprehensive educational KG requires extensive data collection and curation, which is often an ongoing process. Many existing KGs in education are still sparse and contain incomplete information, hindering their effectiveness in providing a holistic view of educational concepts and relationships. Educational KGs aim to encompass various educational concepts, including subjects, topics, learning objectives, and competencies. However, educational data is often fragmented and dispersed across various sources. Some concepts may be well-represented in the KG, while others may have limited or no coverage. This sparsity can result from variations in curricula across institutions, the availability of digital learning resources, and the focus of data collection efforts. Therefore, efforts should be made to integrate data from multiple sources using data integration techniques, data cleaning, and transformation processes. Further, collaboration among educational institutions, publishers, and government agencies can lead to more comprehensive and standardized data sharing. Initiatives like the Learning Resource Metadata Initiative (LRMI)^1^ promote the use of metadata standards to describe educational resources.

**Scalability Challenges**: As the volume of educational content and data continues to grow, scalability becomes a significant challenge. Many of the examined KG construction approaches struggle to keep pace with the ever-expanding educational landscape, resulting in outdated or incomplete knowledge representations. Educational institutions generate vast amounts of data daily, including student records, course materials, research publications, and administrative documents. Constructing a comprehensive KG from this data can be overwhelming, especially for large universities or school districts. Handling the sheer volume of data efficiently is a significant challenge. As educational KGs grow, the number of entities (e.g., courses, students, teachers) and relationships (e.g., prerequisites, enrollment, authorship) in the graph also increases. This expanded graph can become challenging to manage, query, and update, leading to performance issues. To tackle such a challenge, distributed computing frameworks like Apache Hadoop and Apache Spark can be used to process and analyze large volumes of data efficiently. These frameworks can handle parallel processing, making them suitable for KG construction tasks. Also, leveraging semantic indexing and search techniques can enhance query performance in KGs [132]. Implementing semantic search engines can help users find relevant information faster. Cloud-based hosting services provide scalability benefits by allowing dynamic resource allocation based on demand. This can be cost-effective for managing educational KGs [133]. Addressing scalability challenges in educational KGs requires a combination of technological solutions, distributed computing approaches, and optimization techniques. By strategically designing and managing KGs with scalability in mind, educational institutions can unlock the full potential of these knowledge resources for personalised learning, research, and decision-making.

**Educational Semantic Heterogeneity**: Educational KGs often deal with semantically heterogeneous data sources, such as textbooks, research papers, and multimedia content. Integrating these diverse sources into a coherent KG without loss of meaning is a technical challenge. For example, "student performance" might be referred to as "learner achievement" or "academic progress." These variations in terminology can lead to semantic heterogeneity, making it challenging to establish consistent relationships between concepts. Also, multiple languages and cultural contexts may be at play in diverse educational environments. Educational content might need to be represented in different languages, with variations in terminology and cultural nuances. Translating and aligning these diverse representations within a KG can introduce semantic heterogeneity. There is a lack of techniques used in the current approaches to tackle data heterogeneity. Addressing this challenge requires a combination of standardization, alignment, and user-centric design to create more coherent and interoperable knowledge representations in the educational domain.

**Real-time Updates**: Educational knowledge is not static; it evolves with new research, discoveries, and pedagogical trends. Ensuring that KGs are updated in real-time to reflect these changes is a technical demand that requires automated processes. Also, curricula are often adjusted to cater to students' specific needs and progress. For instance, a teacher might modify the syllabus based on the class's learning pace and understanding. Real-time updates enable teachers to reflect these adaptations in the KG, ensuring that students access the most relevant learning materials. However, the examined studies did not consider the temporal dimension into consideration. Most of the current KG construction techniques neglect this vital aspect. Real-time updates in educational KGs are essential for keeping knowledge, curricula, and resources up-to-date in a dynamic educational ecosystem. They support curriculum adaptation, research integration, assessment, resource management, collaboration, and communication. However, addressing challenges related to data volume, quality, security, integration, scalability, and user experience is crucial for the successful implementation of real-time updates in educational KGs.

**Poor Evaluation Techniques:** Developing standardized evaluation metrics for KGs in education is crucial. Assessing the educational effectiveness of KGs often involves subjective factors, such as learner engagement, knowledge retention, and the quality of learning outcomes. These aspects are challenging to quantify and measure objectively, leading to a reliance on user surveys and feedback, which may not provide a complete picture. The examined reports show inadequacy in delivering a solid and proper evaluation of the constructed KGs and their application in downstream tasks. Establishing standardized evaluation metrics, creating openly accessible ground truth datasets, and conducting rigorous, longitudinal studies to measure the impact of KGs on education outcomes are essential steps toward improving the quality and effectiveness of educational KGs.

**Privacy and Security Concerns**: KGs in education often include student records containing personally identifiable information (PII) such as names, addresses, and academic performance. Unauthorized access or data breaches can result in identity theft, fraud, or harassment. Learning analytics over educational KGs collect data on student behaviour, such as online activity, assessment performance, and engagement metrics. This data can be used to create detailed profiles of individuals, raising concerns about surveillance and personal privacy. Another important consideration is the security of the KGs. The construction of the KGs must also consider the risk of insider and outsider threats, whereby individuals with legitimate or illegitimate access misuse data. Proper access controls and monitoring are necessary to mitigate this risk. However, this vital aspect has not been well-studied in the literature. Privacy and security concerns are significant challenges in educational KGs. Institutions must adopt a proactive and multi-faceted approach to address these concerns effectively. This includes technical measures, policy development, and ongoing vigilance to protect sensitive data and ensure compliance with regulations.

## 6 Future directions and research opportunities

The field of education is undergoing a remarkable transformation driven by advances in technology, data analytics, and pedagogical research. As educational institutions and learners adapt to an increasingly digital and interconnected world, there is a growing need for innovative solutions that can enhance teaching and learning outcomes. Future directions and research opportunities in education are poised to shape how we deliver knowledge, personalise learning experiences, and assess educational progress. This section provides a glimpse into the promising avenues of research and development that hold the potential to revolutionize education in the coming years. From personalised learning pathways powered by artificial intelligence to the ethical use of educational data and the integration of emerging technologies, the future of education is both exciting and challenging, offering ample opportunities for scholars, educators, and technologists to make a profound impact on how we acquire and share knowledge.

**Personalised Learning Path**: The use of KGs for personalised learning is still in its early stages, but it has the potential to revolutionize the way that we teach and learn languages. As KGs become more sophisticated and accessible, we can expect to see even more innovative ways to use them to support language learning. Unlike traditional one-size-fits-all approaches, personalised learning leverages KGs to cater to the unique needs, preferences, and progress of each learner. This advanced approach relies on continuous data collection and analysis, considering factors such as a student's prior knowledge, learning style, performance history, and even real-time interactions with educational content. By harnessing the rich web of interconnected knowledge stored in KGs, educational systems can precisely pinpoint knowledge gaps and recommend customized learning resources. For instance, if a student struggles with a specific mathematical concept, the KG can identify the root cause of the difficulty and suggest targeted exercises, videos, or interactive simulations to address it. However, to fully realize the potential of personalised learning paths, challenges related to data privacy, algorithm transparency, and equitable access to technology must be thoughtfully addressed and explored.

**Emerging Trends in KGs and Education:** The education industry is undergoing a profound transformation driven by technological advancements, and the role of KGs has become increasingly prominent. They offer innovative solutions to age-old challenges in education, from personalizing learning experiences to improving content recommendation and supporting lifelong learning. These trends reflect the growing recognition of the value of KGs in enhancing educational processes and outcomes. For example, there is a need to incorporate techniques for building AI-powered virtual tutors; AI-driven virtual tutors and chatbots are leveraging KGs to provide real-time assistance to students. These systems understand student queries, identify knowledge gaps, and offer relevant explanations and resources, enhancing the learning experience. Despite some attempts in this direction [[134], [135], [136]], the incorporation of KG technology in this context is still unexplored.

**Incorporating Large Language Models (LLMs):** The integration of LLMs like GPT-3 and KGs into the field of education holds immense promise, with a multitude of future research opportunities awaiting exploration [137,138]. The coexistence of LLMs and symbolic knowledge representations indeed presents a fascinating landscape for the future of NLP and AI [139]. Therefore, there is a pressing need to develop hybrid models that seamlessly combine LLMs' natural language understanding capabilities with the structured knowledge representation of KGs [140]. This can enable educational systems to understand and generate human-like text and to retrieve and reason over structured knowledge, thereby facilitating more intelligent and context-aware educational assistants. In this context, future research is needed to refine the techniques for automatic KG construction from educational texts, ensuring that this process is scalable and adaptable to various educational domains. Developing methods to enhance the coverage, accuracy, and real-time updates of these KGs is paramount [141]. Moreover, studying the interoperability of KGs from different educational institutions or domains could lead to the creation of global, comprehensive educational KGs. Future research should focus on fine-tuning LLMs for specific educational tasks, such as question-answering, essay grading, or personalised learning path recommendations. These models could be trained on educational KGs to impart domain-specific knowledge and context awareness, thereby enhancing their performance in educational applications. The integration of LLMs and KGs in education opens up exciting possibilities for improving the quality and effectiveness of educational experiences. However, realizing this potential requires concerted research efforts to address technical, ethical, and pedagogical challenges. By developing hybrid models, automating KG construction, ensuring data accuracy, and focusing on task-specific fine-tuning, the future of AI in education can be shaped into a more intelligent and context-aware landscape.

**Cross-Domain KGs for Education:** Cross-Domain KGs are emerging as a transformative force in education, fostering interdisciplinary learning and expanding the horizons of traditional domain-specific knowledge [142]. In education, where learners often encounter complex problems that span multiple subjects, cross-domain KGs serve as invaluable resources. They seamlessly integrate knowledge from diverse domains, breaking down the silos that traditionally separated subjects. This integration opens up new opportunities for holistic and context-aware learning experiences. For example, a biology student studying environmental conservation can effortlessly access related knowledge from ecology, chemistry, and policy-making domains through a cross-domain KG. Moreover, educators can design interdisciplinary courses that leverage these KGs, enabling students to explore real-world challenges that demand multifaceted expertise. However, the development of effective cross-domain KGs in education is not without its challenges. Ensuring data quality, addressing semantic heterogeneity, and maintaining privacy in cross-domain knowledge integration are complex issues that require careful consideration. Nonetheless, the potential benefits of cross-domain KGs in education are immense, promising to enrich learning experiences and prepare students for the complexities of the interconnected world. Therefore, more research is needed to develop advanced techniques like ontology alignment, semantic mapping, and cross-domain concept linking to improve the interoperability of knowledge in KGs. These efforts should also consider applying privacy-preserving techniques and ethical guidelines for handling sensitive information within cross-domain KGs.

## 7 Conclusion

Knowledge Graphs have become a cornerstone in shaping the future of education. They have the capacity to adapt, evolve, and provide customized learning experiences in an increasingly digital and interconnected world. As the education sector continues to embrace these innovative technologies, we stand at the cusp of a transformative era where knowledge is not just acquired but seamlessly interconnected and dynamically personalised for the benefit of learners worldwide. This SLR has provided a comprehensive overview of the diverse applications of KGs in the field of education. Throughout our examination of five primary domains, namely Adaptive and Personalised Learning, Curriculum Design and Planning, Concept Mapping and Visualization, Semantic Search and Questioning Answering, and miscellaneous applications, we witnessed the promise of KGs in delivering tailored and data-driven educational experiences. These domains represent critical areas where KGs are reshaping traditional educational paradigms and empowering educators and learners alike. Our meticulous analysis of the methodologies employed in KG construction and application has shed light on the specific functionalities of these KGs, the knowledge extraction techniques utilized, the nature of the knowledge bases, resource requirements, relevant statistics, evaluation criteria, and limitations. We have identified both the remarkable achievements and the persistent challenges that researchers and practitioners face in this field.

However, it is crucial to acknowledge that our literature review has highlighted several limitations and inadequacies. The lack of standardized formats and ontologies for educational KGs, limited interoperability, sparse and incomplete data, scalability challenges, semantic heterogeneity, real-time updates, poor evaluation techniques, and privacy and security concerns are among the pressing issues that need to be addressed for the widespread and effective use of KGs in education. As we look toward the future, the educational landscape is poised for further transformation. Personalised learning paths driven by artificial intelligence, the incorporation of large language models into KGs, cross-domain KGs for interdisciplinary learning, and real-time updates are just a few of the promising directions for future research and development. Additionally, addressing the limitations and challenges identified in this review will be crucial to realizing the full potential of KGs in education.

## Ethics approval and consent to participate

Not applicable.

## Consent for publication

We give the publisher the permission of the authors to publish the work.

## Availability of data and materials

No data was used for the research described in the article.

### Funding

This work was supported and funded by the Deanship of Scientific Research at Imam Mohammad Ibn Saud Islamic University (IMSIU) (grant number IMSIU-RP23045).

## CRediT authorship contribution statement

**Bilal Abu-Salih:** Writing – review & editing, Writing – original draft, Visualization, Validation, Supervision, Software, Resources, Project administration, Methodology, Investigation, Formal analysis, Data curation, Conceptualization. **Salihah Alotaibi:** Writing – review & editing, Validation, Supervision, Resources, Project administration, Funding acquisition, Formal analysis, Data curation.

## Declaration of competing interest

The authors declare that they have no known competing financial interests or personal relationships that could have appeared to influence the work reported in this paper.

## Footnotes

1 https://www.dublincore.org/about/lrmi/.
